# Supplementary material for: Risk factors for sacrococcygeal pilonidal sinus: a systematic review and meta-analysis supplemented by genetic causal assessment
Source: Front Surg. 2026 Jan 7;12:1718589. doi: 10.3389/fsurg.2025.1718589 (PMC12819706; doi:10.3389/fsurg.2025.1718589)
Supplement: Supplementary file 2 [file Datasheet2.zip › Supplementary Data 2/MR_pipeline_p5e-8/leaveone_plots/ieu-a-93.finngen_R12_L12_PILONIDALCYST.pdf]

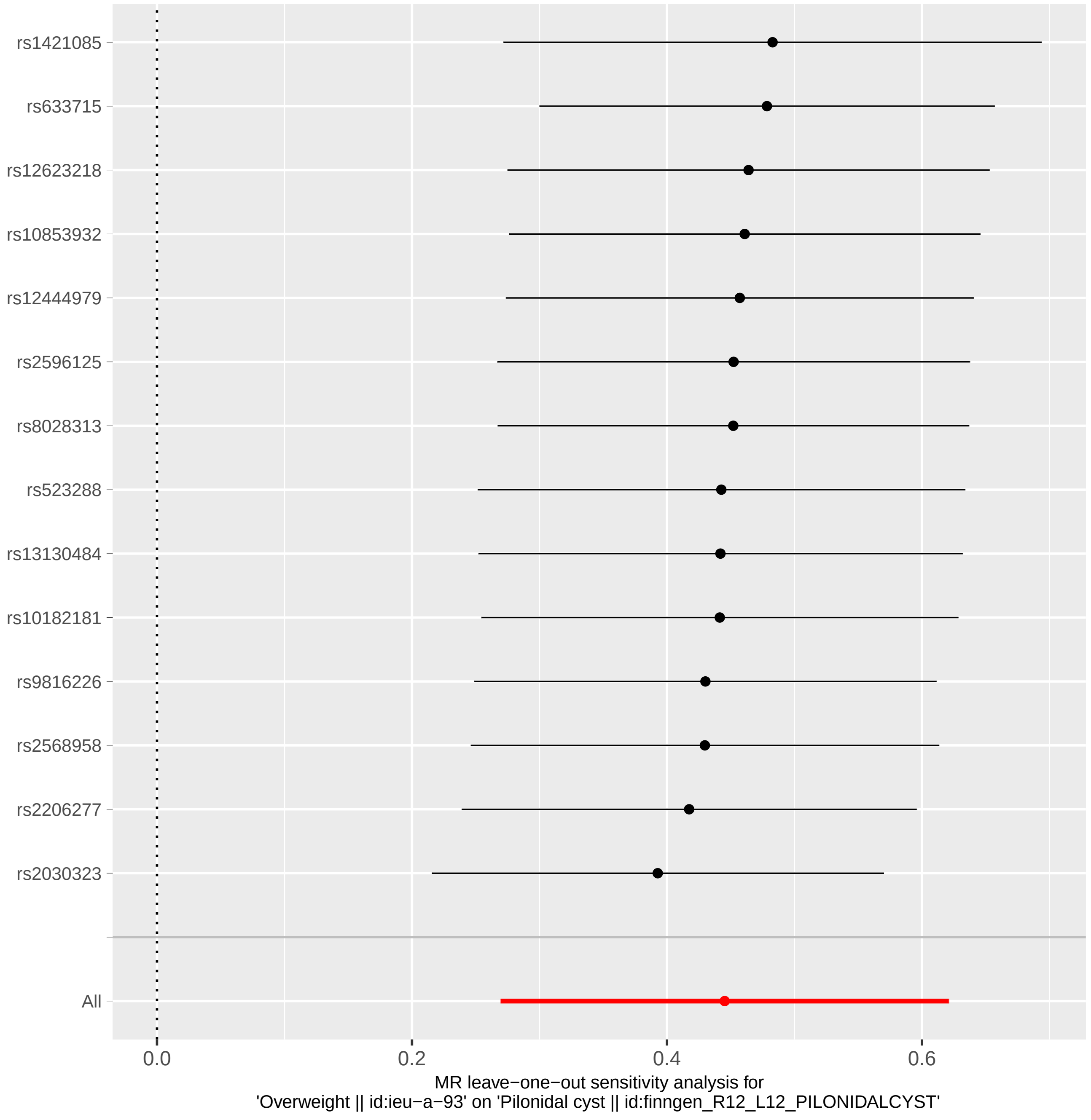
rs1421085
rs633715
rs12623218
rs10853932
rs12444979
rs2596125
rs8028313
rs523288
rs13130484
rs10182181
rs9816226
rs2568958
rs2206277
rs2030323
All
0.0
0.2
0.4
0.6
MR leave−one−out sensitivity analysis for
'Overweight || id:ieu−a−93' on 'Pilonidal cyst || id:finngen_R12_L12_PILONIDALCYST'
